# A longitudinal change of syndecan-1 predicts risk of acute respiratory distress syndrome and cumulative fluid balance in patients with septic shock: a preliminary study

**DOI:** 10.1186/s40560-021-00543-x

**Published:** 2021-03-16

**Authors:** Yuka Kajita, Tsuguaki Terashima, Hisatake Mori, Md. Monirul Islam, Takayuki Irahara, Masanobu Tsuda, Hideki Kano, Naoshi Takeyama

**Affiliations:** 1 Department of Emergency and Critical Care Medicine, Aichi Medical University, Aichi, 480-1195 Japan; 2 Division of Emergency Care, Aichi Medical University, Aichi, 480-1195 Japan

**Keywords:** Glycocalyx, ARDS, Septic shock, Hyperpermeability, Syndecan-1, Cumulative fluid balance

## Abstract

**Background:**

The purpose of this study is to investigate the time course of syndecan-1 (Syn-1) plasma levels, the correlation between Syn-1 and organ damage development, and the associations of Syn-1 level with cumulative fluid balance and ventilator-free days (VFD) in patients with septic shock.

**Methods:**

We collected blood samples from 38 patients with septic shock upon their admission to ICU and for the first 7 days of their stay. Syn-1 plasma level, acute respiratory distress syndrome (ARDS), other organ damage, VFD, and cumulative fluid balance were assessed daily.

**Results:**

Over the course of 7 days, Syn-1 plasma levels increased significantly more in patients with ARDS than in those without ARDS. Patients with high levels of Syn-1 in the 72 h after ICU admission had significantly higher cumulative fluid balance, lower PaO_2_/FiO_2_, and fewer VFD than patients with low levels of Syn-1. Syn-1 levels did not correlate with sequential organ failure assessment score or with APACHE II score.

**Conclusions:**

In our cohort of patients with septic shock, higher circulating level of Syn-1 of cardinal glycocalyx component is associated with more ARDS, cumulative positive fluid balance, and fewer VFD. Measurement of Syn-1 levels in patients with septic shock might be useful for predicting patients at high risk of ARDS.

**Supplementary Information:**

The online version contains supplementary material available at 10.1186/s40560-021-00543-x.

## Background

Sepsis is a life-threatening organ dysfunction caused by infection-induced dysregulation of host responses, which may be complicated by septic shock when circulatory and cellular/metabolic dysfunction occur [1]. Although mortality associated with sepsis has declined owing to better and more intense critical care management settings and new diagnostic and therapeutic methods, it remains a deadly condition [2].

The endothelial glycocalyx is a carbohydrate-rich gel-like layer lining the luminal side of the endothelium surface, composed of syndecan (Syn), hyaluronic acid, chondroitin sulfate, and heparan sulfate [3]. The glycocalyx is the primary physical barrier between blood and the vessel wall, so its damage can have many pathophysiological consequences, such as an increase in vascular permeability, edema, an increase in adhesion of circulating inflammatory cells to the endothelium, acceleration of inflammatory processes, activation of the coagulation cascade, platelet hyperaggregation, and disturbance of microcirculatory flow [4–7], all of which are important determinants of the pathophysiology of sepsis [8, 9]. Loss of vascular integrity has been difficult to assess routinely in the clinical setting because there are no widely applicable tools to measure this process.

Given the pathophysiological implications of glycocalyx degradation, glycocalyx fragments that are shed into the circulation may serve as clinically relevant biomarkers of vascular integrity [10–13]. Glycocalyx degradation as assessed by increased shedding of Syn-1 and hyaluronic acid has been observed in various pathologic states including sepsis [5, 7, 10–13], hemorrhagic shock [7, 14], acute coronary syndrome [7, 15], volume overload [7], and surgical and traumatic injury [14, 16], which have been associated with poor outcome [17]. However, despite the known role of pulmonary endothelial injury and activation in the pathogenesis of acute respiratory distress syndrome (ARDS), the association between glycocalyx damage and the development of ARDS or other organ damage in sepsis is not adequately understood yet. Because most earlier studies involved single-point sample collection upon admission to the emergency room or intensive care unit (ICU), few data exist with which to assess changes in glycocalyx shedding over time and the relationship of these changes to the development of vascular integrity.

We hypothesized that shedding of Syn-1 is accelerated as a result of glycocalyx damage and causes loss of vascular integrity and that sustained pulmonary endothelial damage would contribute to the development of ARDS in patients with septic shock. We sought to (1) determine the time course of changes in Syn-1 and (2) examine the association of these changes with (a) the development of organ damage and scores from the Sequential Organ Failure Assessment (SOFA) and Acute Physiology and Chronic Health Evaluation (APACHE) II and (b) cumulative fluid balance, PaO_2_/FiO_2_, and ventilator-free days (VFD).

## Methods

### Patient selection

This prospective, single-center, observational study was conducted in accordance with the Declaration of Helsinki and was approved by the Institutional Review Board of Aichi Medical University (2017-H341, 2019-H137). This study was performed in a 12-bed, closed-format, mixed medical-surgical emergency ICU in a tertiary referral hospital with 800 beds. Patients were under the direct care of a team of intensivists, subspecialty fellows, and residents regardless of the time of day.

**Fig. 1:**
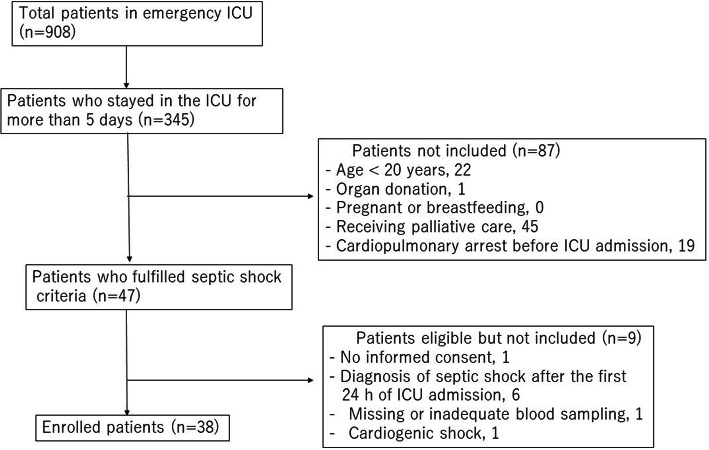
Flowchart summarizing the patient selection procedure in the emergency ICU

Of 908 consecutive patients admitted to the emergency ICU of the Aichi Medical University Hospital between September 2018 and February 2020, we included 38 patients who fulfilled the Sepsis-3 criteria for septic shock upon ICU admission and who stayed in the ICU for more than 5 days. The detailed patient enrollment process is summarized in Fig. 1.

After receiving approval from the Board, written informed consent was obtained from each subject or from a relative or legal representative if direct consent could not be obtained. Septic shock was defined according to the Sepsis-3 criteria, and the diagnosis was confirmed by 2 experienced intensivists [1]. To explain briefly, diagnosis of septic shock required the presence of sepsis, circulatory failure (mean arterial pressure <65 mmHg with norepinephrine at 0.1 μg/kg/min), and a lactate level >2 mmol/L. Infection was diagnosed on the basis of clinical signs of infection and/or culture of microorganisms from suspected foci. The presence of organ dysfunction was defined as an increase of the SOFA score by ≥2 points [18]. All patients were managed according to the 2016 Surviving Sepsis Campaign protocol [19], including fluid resuscitation, use of vasopressors, red cell/platelet transfusion, protective lung strategies (lower tidal volume, lower plateau pressure, and higher positive end-expiratory pressure), and timely initiation of antibiotic therapy.

### Sample measurement

Whole blood samples were collected from 38 patients with septic shock upon emergency ICU admission and for the first 7 days of emergency ICU stay, and at equivalent time points from 15 healthy volunteers (median age 60, and 60% men) with no significant acute or chronic illnesses. Plasma collected from healthy volunteers was used to determine the Syn-1 reference range. Plasma was collected after centrifugation and stored at −70 °C until analysis. Commercially available enzyme-linked immunosorbent assay was used to determine concentrations of Syn-1 (Abcam, Cambridge, UK; lower limit of detection 8 ng/mL) in the plasma samples.

### Clinical data collection

Clinical data including age, sex, comorbidities, chest radiograph findings, clinical time course, cumulative fluid balance, ventilator settings, and treatments were collected from electronic medical records. In addition, the status of clinical severity was assessed with the APACHE II score on admission, and the extent of multiple organ failure was evaluated with the SOFA score daily for the first 3 days. We assessed the central nervous system SOFA score before intubation because it is difficult to assess the exact mental status of patients under sedation. The maximum SOFA score was the highest value reached during the first 3 days. Microbiological and clinical infections were evaluated daily. The daily cumulative fluid balance was computed by subtracting fluid output from fluid intake. Fluid intake included oral, tube feeding, and intravenous intake. Fluid output included urine, drains, bleeding, gauze, and renal replacement amounts. All daily fluid indices (fluid intake, output, and balance) were measured for the first, second, and third days of ICU admission, and the cumulative fluid balance was registered at 72 h for that period. All values were adjusted to the individual’s initial body weight and expressed as ml/kg body weight per 72 h.

The presence of ARDS was assessed daily for the first 3 days according to the Berlin definition, which includes bilateral radiographic infiltrates on chest radiograph, acute onset with worsening respiratory status, and hypoxemia defined as PaO_2_/FiO_2_ <300 mmHg while receiving positive end-expiratory pressure or continuous positive airway pressure of >5 cm H_2_O [20]. Patients with evidence of a primary cardiogenic course for pulmonary edema were not considered to have ARDS. Patients who met criteria for ARDS for at least two consecutive blood gas analyses performed every 6 h were considered to have ARDS; patients who met ARDS criteria at only one blood gas analysis time point or at non-consecutive time points were not considered to have ARDS.

The presence of DIC was assessed daily for the first 3 days according to the Japanese Association for Acute Medicine DIC criteria (see Additional file 1) [21], which include systemic inflammatory response syndrome, platelets, prothrombin time ratio, and fibrinogen degradation product.

### Statistics

Data were collected in MS Windows Office Excel 2013. All statistical analyses were performed using SigmaPlot v14.0 (Systat Software Inc., San Jose, CA, USA) and IBM SPSS v 27 (SPSS Inc, Chicago, Ill, USA). Our study was powered to detect a difference between Syn-1 level and parameters of septic shock, including cumulative fluid balance, VFD, PaO_2_/FiO_2_, catecholamine, and the presence of ARDS. On the basis of mean differences and corresponding standard deviations, we calculated that we needed a sample size of 15 to 38 patients to find a difference in each parameter, with a 2-sided confidence interval of 0.95 and a desired power of 0.8.

Categorical variables are reported as absolute numbers and percentages, and continuous variables are shown as the median with interquartile range (IQR) due to the skewed distribution of most of the parameters. Correlation between plasma Syn-1 level and PaO_2_/FiO_2_ was assessed by a Spearman rank-order correlation analysis. Plasma Syn-1 level, maximum SOFA score, APACHE II score, VFD and cumulative fluid balance, and parameters for organ failure were compared between the high and low Syn-1 groups by the Mann-Whitney rank sum test. The comparison of clinical characteristics of septic shock between ARDS and non-ARDS patients was performed by the Mann-Whitney rank sum or Chi-square test. A threshold of Syn-1 level was defined to 75% IQR of all measured Syn-1 level from septic shock patients. To evaluate whether sepsis-related ARDS significantly changed the overall plasma values of Syn-1 during the period after admission, we used a 2-way repeated measures ANOVA. The distribution of time course changes in plasma Syn-1 trends during the late stage of septic shock and the presence of ARDS were compared by Fisher’s exact test.

## Results

### Patient characteristics

The demographic data of the patients and their illness severity scores calculated in the emergency ICU are summarized in Table 1. All 38 patients (73% men) with a median age of 75 years fulfilled the criteria for diagnosis of septic shock. All patients were vasopressor-dependent at the time of inclusion in the study. The primary sites of infection were intra-abdominal infection in 15, urinary tract infection in 10, and respiratory tract infection in 6. The septic shock cohort had a median maximum SOFA score estimated during the first 3 days of 11 and APACHE II score at admission of 30, indicating high disease severity. The median (IQR) of Syn-1 was significantly higher in patients with septic shock than in healthy controls (265 [168–494] vs 41 [27–65], *P* < 0.001).

**Table 1:** Clinical characteristics of patients with septic shock in the presence or absence of acute respiratory distress syndrome (ARDS)

Characteristic	All septic shock patients (*n*=38) Median (IQR) or *n* (%)	Septic shock patients with ARDS (*n*=20) Median (IQR) or *n* (%)	Septic shock patients without ARDS (*n*=18) Median (IQR) or *n* (%)	*P* value
Age, years	75 (67, 81)	78 (68, 82)	72 (64, 79)	0.396
Male	28 (73)	14 (70)	14 (78)	0.257
ICU stay, days	9 (7, 15)	10 (8, 17)	9 (5, 11)	0.026
Site of infection				
Intra-abdominal	15 (40)	7 (35)	8 (44)	0.793
Respiratory tract	6 (16)	3 (15)	3 (17)	1.0
Urinary tract	10 (26)	6 (30)	4 (22)	0.719
Skin/soft tissue	3 (8)	1 (5)	2 (11)	0.595
Others	4 (9)	3 (15)	1 (6)	0.606
Nonhospital health care-associated infection	11 (29)	6 (30)	5 (28)	0.836
Bacteria				
Gram-positive	14 (37)	7 (35)	7 (39)	0.929
Gram-negative	18 (47)	10 (50)	8 (44)	0.986
Both	4 (11)	2 (10)	2 (11)	1.0
Fungus	2 (5)	1 (5)	1 (6)	1.0
Maximum SOFA^a^	11 (9, 15)	14 (9, 17)	9 (7, 11)	0.202
APACHE II score^b^	30 (26, 34)	32 (28, 35)	30 (26, 31)	0.063
Presence of comorbidities	16 (42)	7 (35)	9 (50)	0.544
Surgical intervention	16 (42)	6 (30)	10 (56)	0.206
Mechanical ventilation	22 (58)	20 (100)	2 (11)	<0.001
ICU deaths	1 (2.6)	1 (5)	0 (0)	1.0
Syn-1, ng/ml	265 (168, 494)	423 (193, 826)	177 (168, 494)	<0.001

Abbreviations: *SOFA*, Sequential Organ Failure Assessment; *APACHE II*, Acute Physiology and Chronic Health Evaluation II; *IQR*, interquartile range; *Syn-1*, syndecan-1; *ARDS*, acute respiratory distress syndrome

^a^SOFA score ranges from 0 to 24

^b^APACHE II score ranges from 0 to 71

*P* values are given for comparison of septic shock between ARDS and non-ARDS patients

### Longitudinal change of Syn-1 level and risk of ARDS

**Fig. 2:**
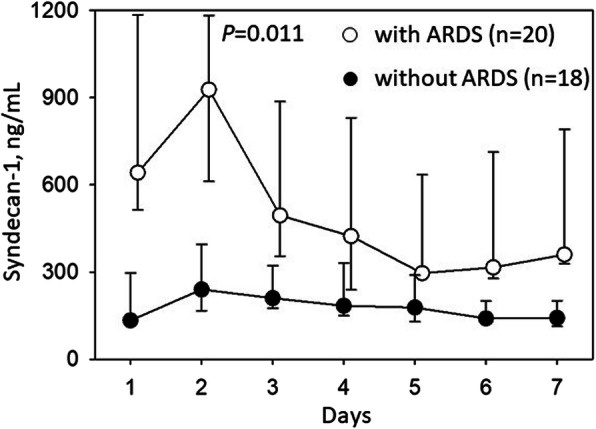
Course of syndecan-1 (Syn-1) levels over 7 days in septic shock patients with (*n* = 20) or without (*n* = 18) acute respiratory distress syndrome (ARDS). Over the course of 7 days, plasma Syn-1 levels were significantly higher in the group with ARDS than in the group without ARDS (2-way repeated measures ANOVA). The data are expressed as medians and 95% CIs

**Fig. 3:**
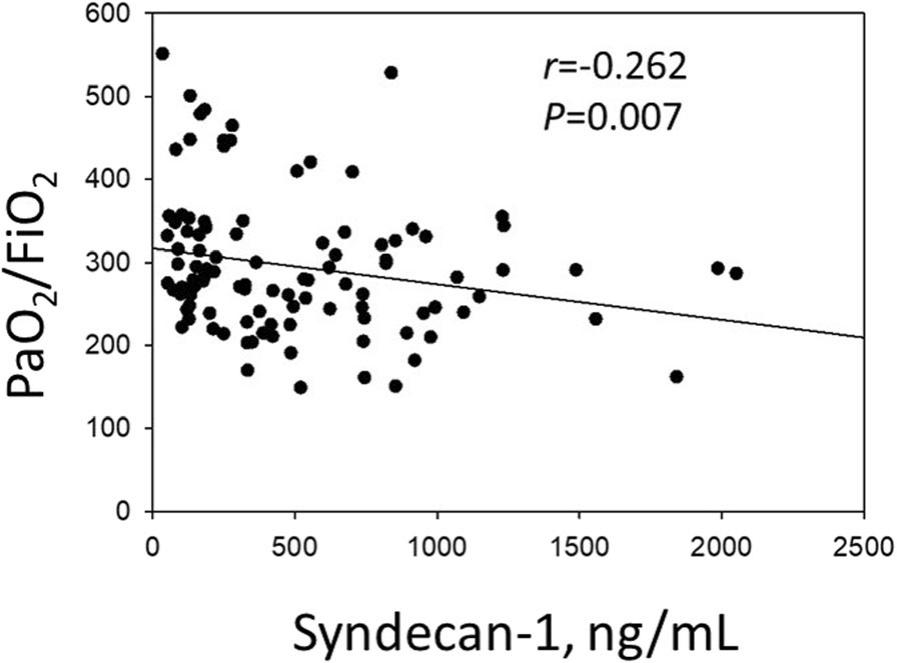
Scattergram of plasma Syn-1 concentration and PaO_2_/FiO_2_ and Spearman rank order correlation analysis in patients with septic shock and secondary acute respiratory distress syndrome (ARDS)

Over the course of 7 days, Syn-1 plasma levels increased significantly more in patients with ARDS than in those without ARDS (Fig. 2). Syn-1 initially increased in patients with ARDS and then decreased, but it consistently remained at a higher level than in patients without ARDS. We found a weak negative correlation between the plasma Syn-1 level and PaO_2_/FiO_2_ in patients with septic shock and secondary ARDS (Fig. 3). Comparing the clinical characteristics of patients with ARDS and non-ARDS, there are no significant differences in age, gender, site of infection, pathogen, severity of illness, surgical intervention, ICU death, maximum SOFA, APACHE II, and comorbidities. A significant difference between these groups were seen for ICU stay, mechanical ventilation, and Syn-1 level (Table 1).

### Distribution of Syn-1 levels of patients with septic shock

**Fig. 4:**
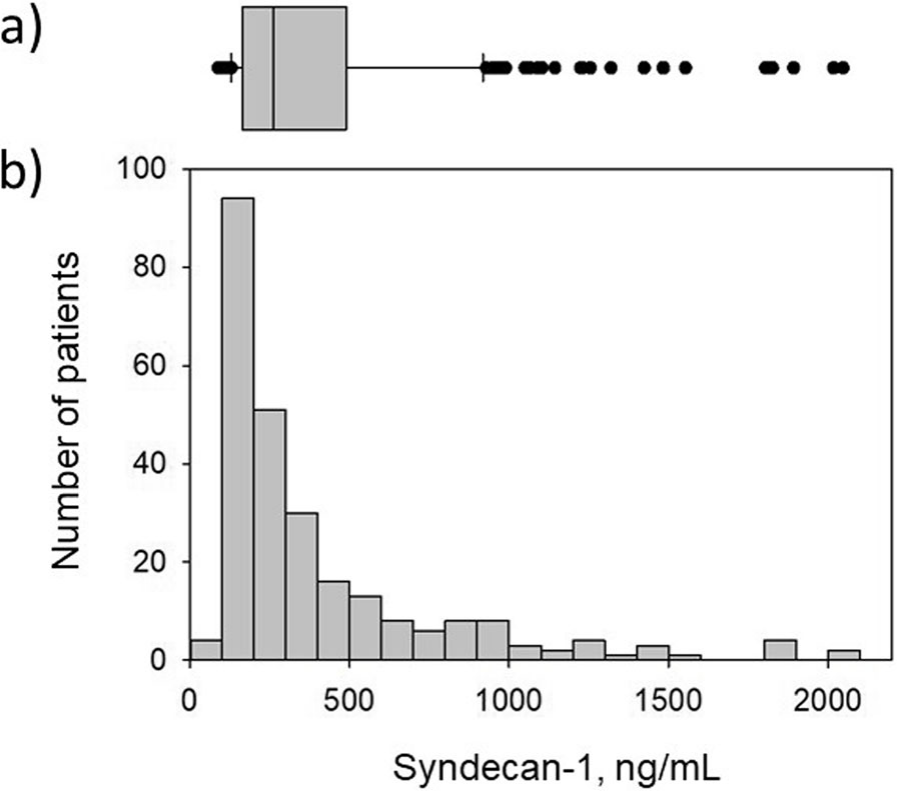
Distribution of Syn-1 levels in patients with septic shock (*n* = 38). **a** Box plot is shown with median (line inside box), 25th and 75th percentiles (left and right lines of box), and range (whiskers). Any data not included between the whiskers are plotted as outliers (small circles). **b** Histogram is plotted with 258 samples collected from patients with septic shock

The distribution of Syn-1 levels is shown in Fig. 4a and b; the median level was 265 ng/mL (IQR, 168 to 494 ng/mL). Patients with septic shock were divided into 2 groups by using the upper (third) quartile (Q3) as the cutoff value. Patients with a Syn-1 level below Q3 (<494 ng/mL) were defined as the low Syn-1 group, and those with a Syn-1 level above Q3 (**>** 494 ng/mL) were defined as the high Syn-1 group. Moreover, the highest Syn-1 level measured 1 to 3 days after ICU admission was defined as the Syn-1 level in the early stage of septic shock, and the highest Syn-1 level measured 5 to 7 days after admission was defined as the Syn-1 level in the late stage of septic shock. According to the Q3 threshold, in the early stage of septic shock, 21 of the 38 patients were assigned to the high Syn-1 group and 17 to the low Syn-1 group (Table 2). In the late stage of septic shock, 11 of the 38 patients were assigned to the high Syn-1 group and 27 to the low Syn-1 group (Table 2).

**Table 2:** Plasma syndecan-1 (Syn-1) level and organ failure

Characteristics	Syn-1 in early stage of septic shock			Syn-1 in late stage of septic shock		
	High (*n* = 21)	Low (*n* = 17)	*P* value	High (*n* = 11)	Low (*n* = 27)	*P* value
Organ failure						
Creatinine, median (IQR), mg/dL	2.2 (1.3, 4)	1.1 (0.7, 2.5)	0.119	1.9 (1.0, 4.0)	1.9 (0.8, 2.8)	0.669
DIC score, median (IQR)	6 (5, 8)	5.5 (3.3, 7.8)	0.151	6 (4, 8)	6 (5, 8)	0.945
Total bilirubin, median (IQR), mg/dL	1.78 (1.20, 3.27)	1.19 (0.83, 1.68)	0.051	1.94 (1.17, 3.46)	1.3 (0.79, 2.03)	0.113
Glasgow coma scale, median (IQR)	14 (6.8, 15)	14.5 (12, 15)	0.235	14.5 (9, 15)	14 (8.8, 15)	0.578
Norepinephrine dose, median (IQR), mcg/kg/min	0.6 (0.4, 0.8)	0.4 (0.2, 0.5)	0.063	0.6 (0.3, 0.8)	0.4 (0.3, 0.6)	0.14
PaO_2_/FiO_2_, median (IQR)	204 (160, 350)	340 (248, 420)	0.032	194 (161, 274)	330 (204, 420)	0.032
ARDS, *n* (%)	15/21 (71)	5/17 (29)	0.01	10/11 (91)	10/27 (37)	0.006
Cumulative fluid balance, median (IQR), ml/kg/d	172 (124, 239)	120 (42, 154)	0.003	157 (132, 220)	134 (76.6, 198)	0.221
Ventilator-free days, median (IQR)	22 (15, 25)	25 (23, 28)	0.025	23 (19, 25)	25 (20, 28)	0.305
APACHE II^a^, median (IQR)	31 (28, 35)	27.5 (24.3, 31)	0.082	30 (26, 34)	30 (26, 35)	0.636
Maximum SOFA^b^, median (IQR)	12 (10, 17)	9 (7, 15)	0.071	15 (9, 15)	11 (7.8, 15)	0.229
Syn-1, median (IQR), ng/mL	1022 (612, 1473)	253 (168, 415)	<0.001	805 (546, 899)	177 (142, 250)	<0.001

Abbreviations: *APACHE II*, Acute Physiology and Chronic Health Evaluation II; *IQR*, interquartile range; *DIC*, disseminated intravascular coagulation; *ARDS*, acute respiratory distress syndrome; *Syn-1*, syndecan-1

^a^APACHE II score ranges from 0 to 71

^b^Maximum SOFA score ranges from 0 to 24

In both scales, lower scores indicate better organ function. *P* values are given for comparison of high and low Syn-1 groups

### Plasma Syn-1 levels and organ failure

In the early stage of septic shock, the median Syn-1 level was 4 times higher in the high Syn-1 group than in the low Syn-1 group (Table 2), and in the late stage of septic shock, it was 4.5 times higher in the high Syn-1 group than in the low Syn-1 group (Table 2). When comparing organ damage between groups, we found that in the early stage of septic shock, patients in the high Syn-1 group had higher cumulative fluid balance, lower PaO_2_/FiO_2_, more ARDS, and fewer VFD than patients in the low Syn-1 group (Table 2). Other organ damage parameters, including creatinine, Glasgow coma scale, total bilirubin, DIC score, and norepinephrine dose, did not differ significantly between these groups (Table 2). Maximum SOFA scores in the 72 h after ICU admission and APACHE II scores on admission were not significantly different between the two groups (Table 2). In the late stage of septic shock, patients in the high Syn-1 group showed significantly more ARDS than patients in the low Syn-1 group. Because most of our patients with septic shock (97%) survived, we could not explore whether Syn-1 levels are correlated with 30-day mortality.

### Time course changes of plasma Syn-1 levels and ARDS

The patients assigned to the high Syn-1 group in the early stage of septic shock showed 2 distinct Syn-1 trends during the late stage of septic shock: persistently high levels (> 494 ng/ml) throughout 7 days (Group I) or a high peak (> 494 ng/mL) in the first 3 days followed by a progressive decrease (Group II). Of the 21 patients in the high Syn-1 group in the early stage of septic shock, 10 were classed as group I and 11 as group II. All patients in group I had ARDS, whereas only 5 of the 11 patients in group II had ARDS. ARDS was more frequent in patients with high levels of Syn-1 throughout 7 days than in patients with a high peak in the first 3 days followed by a progressive decrease (Fisher’s exact test, *P* = 0.012).

## Discussion

The main finding of the present study is that persistently high plasma Syn-1 levels in patients with septic shock are associated with ARDS and reduction of PaO_2_/FiO_2_. High levels of plasma Syn-1 in the early stage of septic shock are associated with positive cumulative fluid balance, and lower VFD and PaO_2_/FiO_2_. These results suggest that septic shock patients with high circulating Syn-1 level may represent a cohort at particular risk for ARDS.

Recent experimental evidence in rodents showed that the endothelial glycocalyx is reduced by shedding after administration of lipopolysaccharide (LPS) or tumor necrosis factor [22–24]. The disruption of the pulmonary endothelial glycocalyx causes neutrophil-endothelial interaction and fluid and protein extravasation; accordingly, such disruption may mediate LPS-induced ARDS in animal models [24]. In a model of LPS-induced lung injury, intravital [4] and electron microscopy [25] demonstrated a severe disruption and peeling away of moss-like capillary endothelial glycocalyx, accompanied by a reduction in pulmonary Syn-1 levels and an increase in vascular permeability. A recent clinical study showed that endothelial glycocalyx shedding was found in ARDS after flu syndrome [26]. However, there is a lack of studies investigating whether endothelial glycocalyx degradation contributes to ARDS development in patients with bacterial sepsis. The present study shows that the extent of glycocalyx disruption (as assessed by elevated plasma Syn-1 levels in 72 h after ICU admission) is closely related to the development of septic-induced ARDS. Our control values for serum Syn-1 concentrations are consistent with data reported by others [16]. Our finding supports a previous report by Murphy et al. [27], which showed that endothelial glycocalyx degradation assessed by plasma Syn-1 collected on day 2 was associated with the development of secondary ARDS caused by non-pulmonary sepsis. The authors concluded that the contribution of endothelial glycocalyx loss in the pathogenesis of ARDS would be less prominent in primary ARDS than secondary ARDS. Because most (84%) of our patients with septic shock had secondary ARDS, we could not confirm whether different causes of ARDS were correlated differently to the extent of endothelial glycocalyx damage.

The second important finding of the present study is that the relationship between high Syn-1 level over the 7 days after ICU admission and illness severity in early stage of septic shock were not identified except for pulmonary disturbances. Some previous studies in patients with sepsis showed a significant association between plasma Syn-1 level and SOFA score [17], hypocoagulability [10], APACHE II score [17], and development of acute kidney injury [28], whereas other studies showed no significant correlations of Syn-1 with SOFA score [29], noradrenaline infusion, [30] or the Simplified Acute Physiology Score II [29]. The discordance between these results could be attributed to differences in the patients’ backgrounds and comorbidities, since Syn-1 level can be elevated in a range of chronic diseases including heart failure [31], chronic kidney disease [32], and diabetes mellitus [33], which are known to worsen organ failure or its outcomes [34]. The difference in the timings of sample collection may also account for the different findings because almost all previous studies measured Syn-1 at only one time point.

The third important finding of the present study is that the sustained high levels of plasma Syn-1 (as assessed by elevated plasma Syn-1 levels in the early and late stages of septic shock) were closely related to the presence of ARDS. Sustained high levels of plasma Syn-1 suggest a pathological condition caused not only by the continuous shedding of the endothelial glycocalyx but also by an impairment of glycocalyx reconstitution. The resulting delay of glycocalyx reconstitution exacerbates and prolongs the damage to vascular integrity [35, 36].

Because this positive cumulative fluid balance is mainly caused by systemic vascular hyperpermeability (a characteristic finding in patients with septic shock), inflammatory injury to the glycocalyx as assessed by high levels of circulating Syn-1 would be linked to the increase in vascular permeability. Recent literature showed that increased cumulative fluid balance, which leads to increased pulmonary edema and loss of aerated lung tissue, is associated with fewer VFD, lower PaO_2_/FiO_2_, and ARDS [37, 38]. A retrospective review of the Fluid and Catheter Treatment Trial also showed that a negative cumulative fluid balance was associated with more VFD [39]. Our results are consistent with these previous studies. However, further studies are needed to clarify the relation between the development of vascular hyperpermeability during septic shock and the increased levels of circulating Syn-1.

Some limitations of this study should be considered. First, the study was a single-center study with a limited number of patients, which means that our results require confirmation by a larger investigation. Second, this study showed limited analysis of glycocalyx components and so does not allow independent evaluation of the suggested cause-and-effect relationship. To establish potential causality and improve our understanding of the interaction between the glycocalyx, vascular integrity, and lung injury in patients with septic shock, a direct histological analysis of pulmonary specimens is necessary. Third, we assayed circulating levels of glycocalyx components from human plasma samples. While we know that the major source of this protein is the endothelium, some of these glycocalyx components are also present on epithelial cells [40]. It is therefore difficult to ascertain the origin of the circulating glycocalyx components.

## Conclusions

This study shows that higher circulating levels of Syn-1 during the first 7 days of ICU stay are associated with sepsis-related ARDS. Results showed that higher circulating levels of Syn-1 in the 72 h after ICU admission was closely related to positive cumulative fluid balance, and decreased VFD and PaO_2_/FiO_2_. Measurement of Syn-1 levels in patients with septic shock might be useful for predicting patients at high risk of ARDS.

## Supplementary Information

**Additional file 1:.** Title of data: Japanese Association for Acute Medicine (JAAM) Disseminated Intravascular Coagulation (DIC) diagnostic criteria.

## Data Availability

All data generated and/or analyzed during this study are available from the corresponding author on reasonable request.

## Abbreviations

ARDS: Acute respiratory distress syndrome
APACHE II: Acute Physiology and Chronic Health Evaluation II
DIC: Disseminated intravascular coagulation
ICU: Intensive care unit
IQR: Interquartile range
LPS: Lipopolysaccharide
SOFA: Sequential Organ Failure Assessment
Syn-1: Syndecan-1
VFD: Ventilator-free days

## References

[1] Singer M, Deutschman CS, Seymour CW, Shankar-Hari M, Annane D, Bauer M, Bellomo R, Bernard GR, Chiche JD, Coopersmith CM, Hotchkiss RS, Levy MM, Marshall JC, Martin GS, Opal SM, Rubenfeld GD, van der Poll T, Vincent JL, Angus DC (2016). The Third International Consensus Definitions for Sepsis and Septic Shock (Sepsis-3). JAMA..

[2] Pruinelli L, Westra BL, Yadav P, Hoff A, Steinbach M, Kumar V, Delaney CW, Simon G (2018). Delay within the 3-hour surviving sepsis campaign guideline on mortality for patients with severe sepsis and septic shock. Crit Care Med.

[3] Weinbaum S, Tarbell JM, Damiano ER (2007). The structure and function of the endothelial glycocalyx layer. Annu Rev Biomed Eng.

[4] Schmidt EP, Yang Y, Janssen WJ, Gandjeva A, Perez MJ, Barthel L, Zemans RL, Bowman JC, Koyanagi DE, Yunt ZX, Smith LP, Cheng SS, Overdier KH, Thompson KR, Geraci MW, Douglas IS, Pearse DB, Tuder RM (2012). The pulmonary endothelial glycocalyx regulates neutrophil adhesion and lung injury during experimental sepsis. Nat Med.

[5] Chelazzi C, Villa G, Mancinelli P, De Gaudio AR, Adembri C (2015). Glycocalyx and sepsis-induced alterations in vascular permeability. Crit Care.

[6] Colbert JF, Schmidt EP (2016). Endothelial and microcirculatory function and dysfunction in sepsis. Clin Chest Med.

[7] Pillinger NL, Kam PCA (2017). Endothelial glycocalyx: basic science and clinical implications. Anaesth Intensive Care.

[8] De Backer D, Cortes DO, Donadello K, Vincent JL (2014). Pathophysiology of microcirculatory dysfunction and the pathogenesis of septic shock. Virulence..

[9] De Backer D, Creteur J, Preiser JC, Dubois MJ, Vincent JL (2002). Microvascular blood flow is altered in patients with sepsis. Am J Respir Crit Care Med.

[10] Ostrowski SR, Haase N, Muller RB, Moller MH, Pott FC, Perner A (2015). Association between biomarkers of endothelial injury and hypocoagulability in patients with severe sepsis: a prospective study. Crit Care.

[11] Uchimido R, Schmidt EP, Shapiro NI (2019). The glycocalyx: a novel diagnostic and therapeutic target in sepsis. Crit Care.

[12] Iba T, Levy JH (2019). Derangement of the endothelial glycocalyx in sepsis. J Thromb Haemost.

[13] Rovas A, Seidel LM, Vink H, Pohlkötter T, Pavenstädt H, Ertmer C, Hessler M, Kümpers P (2019). Association of sublingual microcirculation parameters and endothelial glycocalyx dimensions in resuscitated sepsis. Crit Care.

[14] Halbgebauer R, Braun CK, Denk S, Mayer B, Cinelli P, Radermacher P, Wanner GA, Simmen HP, Gebhard F, Rittirsch D, Huber-Lang M (2018). Hemorrhagic shock drives glycocalyx, barrier and organ dysfunction early after polytrauma. J Crit Care.

[15] Dekker NAM, Veerhoek D, Koning NJ, van Leeuwen ALI, Elbers PWG, van den Brom CE (2019). Postoperative microcirculatory perfusion and endothelial glycocalyx shedding following cardiac surgery with cardiopulmonary bypass. Anaesthesia..

[16] Rahbar E, Cardenas JC, Baimukanova G, Usadi B, Bruhn R, Pati S, Ostrowski SR, Johansson PI, Holcomb JB, Wade CE (2015). Endothelial glycocalyx shedding and vascular permeability in severely injured trauma patients. J Transl Med.

[17] Anand D, Ray S, Srivastava LM, Bhargava S (2016). Evolution of serum hyaluronan and syndecan levels in prognosis of sepsis patients. Clin Biochem.

[18] Vincent JL, Moreno R, Takala J, Willatts S, De Mendonça A, Bruining H (1996). The SOFA (Sepsis-related Organ Failure Assessment) score to describe organ dysfunction/failure. On behalf of the Working Group on Sepsis-Related Problems of the European Society of Intensive Care Medicine. Intensive Care Med.

[19] Rhodes A, Evans LE, Alhazzani W, Levy MM, Antonelli M, Ferrer R, Kumar A, Sevransky JE, Sprung CL, Nunnally ME, Rochwerg B, Rubenfeld GD, Angus DC, Annane D, Beale RJ, Bellinghan GJ, Bernard GR, Chiche JD, Coopersmith C, de Backer DP, French CJ, Fujishima S, Gerlach H, Hidalgo JL, Hollenberg SM, Jones AE, Karnad DR, Kleinpell RM, Koh Y, Lisboa TC, Machado FR, Marini JJ, Marshall JC, Mazuski JE, McIntyre LA, McLean AS, Mehta S, Moreno RP, Myburgh J, Navalesi P, Nishida O, Osborn TM, Perner A, Plunkett CM, Ranieri M, Schorr CA, Seckel MA, Seymour CW, Shieh L, Shukri KA, Simpson SQ, Singer M, Thompson BT, Townsend SR, van der Poll T, Vincent JL, Wiersinga WJ, Zimmerman JL, Dellinger RP (2017). Surviving sepsis campaign: international guidelines for management of sepsis and septic shock: 2016. Intensive Care Med.

[20] Definition Task Force ARDS, Ranieri VM, Rubenfeld GD, Thompson BT, Ferguson ND, Caldwell E, Fan E (2012). Acute respiratory distress syndrome the Berlin definition. JAMA..

[21] Gando S, Iba T, Eguchi Y, Ohtomo Y, Okamoto K, Koseki K, Mayumi T, Murata A, Ikeda T, Ishikura H, Ueyama M, Ogura H, Kushimoto S, Saitoh D, Endo S, Shimazaki S, Japanese Association for Acute Medicine Disseminated Intravascular Coagulation (JAAM DIC) Study Group (2006). A multicenter, prospective validation of disseminated intravascular coagulation diagnostic criteria for critically ill patients: comparing current criteria. Crit Care Med.

[22] Huang L, Zhang X, Ma X, Zhang D, Li D, Feng J, Pan X, Lü J, Wang X, Liu X (2018). Berberine alleviates endothelial glycocalyx degradation and promotes glycocalyx restoration in LPS-induced ARDS. Int Immunopharmacol.

[23] Wiesinger A, Peters W, Chappell D, Kentrup D, Reuter S, Pavenstädt H (2013). Nanomechanics of the endothelial glycocalyx in experimental sepsis. PLoS One.

[24] Liu XY, Xu HX, Li JK, Zhang D, Ma XH, Huang LN, Lü JH, Wang XZ (2018). Neferine protects endothelial glycocalyx via mitochondrial ROS in lipopolysaccharide-induced acute respiratory distress syndrome. Front Physiol.

[25] Inagawa R, Okada H, Takemura G, Suzuki K, Takada C, Yano H, Ando Y, Usui T, Hotta Y, Miyazaki N, Tsujimoto A, Zaikokuji R, Matsumoto A, Kawaguchi T, Doi T, Yoshida T, Yoshida S, Kumada K, Ushikoshi H, Toyoda I, Ogura S (2018). Ultrastructural alteration of pulmonary capillary endothelial glycocalyx during endotoxemia. Chest..

[26] Benatti MN, Fabro AT, Miranda CH (2020). Endothelial glycocalyx shedding in the acute respiratory distress syndrome after flu syndrome. J Intensive Care.

[27] Murphy LS, Wickersham N, McNeil JB, Shaver CM, May AK, Bastarache JA (2017). Endothelial glycocalyx degradation is more severe in patients with non-pulmonary sepsis compared to pulmonary sepsis and associates with risk of ARDS and other organ dysfunction. Ann Intensive Care.

[28] Puskarich MA, Cornelius DC, Tharp J, Nandi U, Jones AE (2016). Plasma syndecan-1 levels identify a cohort of patients with severe sepsis at high risk for intubation after large-volume intravenous fluid resuscitation. J Crit Care.

[29] Ostrowski SR, Berg RMG, Windeløv NA, Meyer MAS, Plovsing RR, Møller K, Johansson PI (2013). Coagulopathy, catecholamines, and biomarkers of endothelial damage in experimental human endotoxemia and in patients with severe sepsis: a prospective study. J Crit Care.

[30] Johansson PI, Haase N, Perner A, Ostrowski SR (2014). Association between sympathoadrenal activation, fibrinolysis, and endothelial damage in septic patients: a prospective study. J Crit Care.

[31] Neves FM, Meneses GC, Sousa NE, Menezes RR, Parahyba MC, Martins AM (2015). Syndecan-1 in acute decompensated heart failure -association with renal function and mortality. Circ J.

[32] Padberg JS, Wiesinger A, di Marco GS, Reuter S, Grabner A, Kentrup D, Lukasz A, Oberleithner H, Pavenstädt H, Brand M, Kümpers P (2014). Damage of the endothelial glycocalyx in chronic kidney disease. Atherosclerosis..

[33] Wang JB, Guan J, Shen J, Zhou L, Zhang YJ, Si YF, Yang L, Jian XH, Sheng Y (2009). Insulin increases shedding of syndecan-1 in the serum of patients with type 2 diabetes mellitus. Diabetes Res Clin Pract.

[34] Esper AM, Moss M, Lewis CA, Nisbet R, Mannino DM, Martin GS (2006). The role of infection and comorbidity: factors that influence disparities in sepsis. Crit Care Med.

[35] Yang Y, Haeger SM, Suflita MA, Zhang F, Dailey KL, Colbert JF, Ford JA, Picon MA, Stearman RS, Lin L, Liu X, Han X, Linhardt RJ, Schmidt EP (2017). Fibroblast growth factor signaling mediates pulmonary endothelial glycocalyx reconstitution. Am J Respir Cell Mol Biol.

[36] Rizzo AN, Dudek SM (2017). Endothelial glycocalyx repair: building a wall to protect the lung during sepsis. Am J Respir Cell Mol Biol.

[37] van Mourik N, Metske HA, Hofstra JJ, Binnekade JM, Geerts BF, Schultz MJ, Vlaar APJ (2019). Cumulative fluid balance predicts mortality and increases time on mechanical ventilation in ARDS patients: an observational cohort study. PLoS One.

[38] Wiedemann HP, Wheeler AP, Bernard GR, Thompson BT, Hayden D, de Boisblanc B, National Heart, Lung, and Blood Institute Acute Respiratory Distress Syndrome (ARDS) Clinical Trials Network (2006). Comparison of two fluid-management strategies in acute lung injury. N Engl J Med.

[39] Rosenberg AL, Dechert RE, Park PK, Bartlett RH (2009). Association of cumulative fluid balance on outcome in acute lung injury: a retrospective review of the ARDSnet tidal volume study cohort. J Intensive Care Med.

[40] Ochs M, Hegermann J, Lopez-Rodriguez E, Timm S, Nouailles G, Matuszak J, Simmons S, Witzenrath M, Kuebler WM (2020). On top of the alveolar epithelium: surfactant and the glycocalyx. Int J Mol Sci.

