# Supplementary material for: A longitudinal change of syndecan-1 predicts risk of acute respiratory distress syndrome and cumulative fluid balance in patients with septic shock: a preliminary study
Source: J Intensive Care. 2021 Mar 16;9:27. doi: 10.1186/s40560-021-00543-x (PMC7962080; doi:10.1186/s40560-021-00543-x)
Supplement: Supplementary file 1 — Additional file 1:. Title of data: Japanese Association for Acute Medicine (JAAM) Disseminated Intravascular Coagulation (DIC) diagnostic criteria. [file 40560_2021_543_MOESM1_ESM.docx]

**Additional file 1.**

**Japanese Association for Acute Medicine (JAAM) Disseminated Intravascular Coagulation (DIC) diagnostic criteria**

|  | Score |
| --- | --- |
| Systemic inflammatory response syndrome criteria |  |
| ≥3 | 1 |
| 0–2 | 0 |
| Platelet count (×10^9^/L)  <80 or >50% decrease within 24 hours | 3 |
| ≥80 and <120, or 30% decrease within 24 hours  ≥120  Prothrombin time ratio  ≥1.2 | 1  0  1 |
| <1.2 | 0 |
| Fibrin/fibrinogen degradation products (FDP) (μg/mL)* |  |
| ≥25 | 3 |
| ≥10 and <25 | 1 |
| <10 | 0 |
| Diagnosis |  |
| ≥4 points | JAAM-DIC |

*: JAAM-DIC is also calculated by D-dimer.
